# Deferiprone ameliorates cisplatin induced peripheral neurotoxicity via ferritinophagy adjustment

**DOI:** 10.1038/s41598-025-87628-x

**Published:** 2025-02-06

**Authors:** Hanan Seddiek, Mira Hanna, Amany Elsayed Mohamed Hamoud, Marawan Abd Elbaset, Ahmed M. A. Akabawy, Mohamed Zakaria Kotb, Mohamed Mansour Khalifa

**Affiliations:** 1 Department of Medical Physiology, Faculty of Medicine, Kasr Al-Ainy, Cairo University, Cairo, Egypt; 2 Department of Anatomy and Embryology, Faculty of Medicine, Kasr Al-Ainy, Cairo University, Cairo, Egypt; 3 Department of Pharmacology, Medical Research and Clinical Studies Institute, National Research Centre, Cairo, Egypt; 4 Department of Neurology, Indiana University School of Medicine, Indianapolis, IN 46202 USA; 5 Department of Biochemistry and Molecular Biology, Faculty of Pharmacy, Helwan University, 11795 Cairo, Egypt; 6 Department of Medical Physiology, College of Medicine, King Saud University, Riyadh, Kingdom of Saudi Arabia

**Keywords:** Cisplatin, Neurotoxicity, Deferiprone, Ferritinophagy, Ferroptosis, Biochemistry, Cancer, Neuroscience, Physiology, Medical research, Neurology

## Abstract

Cisplatin-induced neurotoxicity is one of the limiting factors to its use especially in tumors that demand high drug dosage. One of the Cisplatin pathways is ferritinophagy which may end up in ferroptosis. So, we aimed to use iron chelator as a new strategy based on an anti-ferroptotic mechanism and to evaluate its neuroprotective effect against polyneuropathy in Cisplatin-treated rats. Twenty-four male Wistar albino rats were arranged into four groups: (I) Control group, rats were given vehicle; (II) Def group, rats received deferiprone (200 mg/kg orally once daily for 10 days); (III) Cis group, rats were injected by Cis 2 mg/Kg once daily for 3 consecutive days i.p.; and (IV) Cis + Def group, rats received deferiprone (200 mg/kg orally once daily for 10 days, rats were injected with Cis in the 4th, 5th, and 6th days). Cis increased and upregulated ferritinophagy inducers significantly including MDA, NCOA4, and IREB1 as compared to the control group. On the other hand, GSH, GPX4, SLCA11 and FTH1 were decreased and down regulated significantly compared to the control group. In addition to significant deterioration in the histopathological and immunological nerve tissue assessment using silver stain and PNCA. Embracing the cisplatin dosage with deferiprone reversed cisplatin-induced neuropathy, in which the physiological function significantly improved along with the immune and histopathology of nerve tissue. This was accompanied by down regulation of ferritinophagy inducers and enhancing ferritinophagy inhibitors. The current results concluded that rapping cisplatin with deferiprone can mitigate neurotoxicity induced by cisplatin in experimental animals through ferritinophagy pathway adjustment.

**Supplementary Information:**

The online version contains supplementary material available at 10.1038/s41598-025-87628-x.

## 1. Introduction

Cisplatin is the first and most effective platinum-based chemotherapy widely used in the treatment of a lot of hazardous tumors such as testicular, ovarian, cervical, breast, bladder, lung, mesothelioma, hepatic and brain tumors. Resistance of aggressive tumors with a defensive program to chemotherapy leads to the use of high doses of cisplatin and combination of antineoplastic drugs. This results in many considerable side effects that may interfere with the use of cisplatin despite its success, to name a few, nephrotoxicity, neurotoxicity, hepatotoxicity, ototoxicity, and hemolytic anemia^1–4^.

Cisplatin is able to bind with the purine bases on the DNA and intervene with DNA repair mechanisms resulting in DNA damage. In addition, other molecular mechanisms include induction of oxidative stress in the form of reactive oxygen species (ROS) production and lipid peroxidation that may lead to apoptosis and necrosis. Also, cellular death can be due to autophagy that includes sequestrated cytoplasmic protein aggregates and weary organelles to be degraded by lysosomal action^1,5^.

Ferroptosis, an expression introduced 12 years ago, is one of the non-apoptotic iron-dependent regulated cell death pathways that can be induced by the anti-tumor activity of cisplatin. So, ferroptosis might play a role in cancer as well. Cisplatin can lead to ferroptosis through inducing ferritinophagy that increases intracellular free iron levels by degradation of ferritin (iron storage protein) by autophagy/lysosomal mechanism. Increased iron level leads to excess ROS accumulation with cis treatment inducing ferroptosis under stressful cellular conditions. This is due to the accumulation of iron-dependent ROS, depletion of reduced glutathione and the inactivation of glutathione peroxidase that has been caused by cisplatin^6–10^.

Nerve toxicity is one of the severe complications of cisplatin chemotherapy in 30–50% of patients in which chronic peripheral sensory neuropathy commonly occurs with a high cumulative dose of cisplatin^4,11^. In-vitro and in-vivo studies showed that the underlying mechanism of neurotoxicity might be due to reduced nerve growth factor, brain-derived neurotrophic factor and neurotrophin-3^12–15^ or mitochondrial cell damage^16^. In addition to the previously stated mechanism of action of cisplatin, ferritinophagy might have been one of those mechanisms.

Therefore, Iron chelation may play a pivotal role in adjusting ferroptosis. Many studies showed that iron chelators can ameliorate the effects of iron toxicity in cell and animal models of diseases. As well they demonstrated the ability of cell-permeable iron chelator to prevent ferroptosis^17–21^. The concern could be attracted more towards the identification and understanding of the biochemical and cellular mechanisms of neuroprotectants than that of toxicity^22–24^. There is a detected relation between iron accumulation, ROS, and ferroptosis in which ferroptosis may be a result of accumulated ROS, induced by impaired iron export. So, it has been suggested that iron chelators, anti-oxidants, and inhibitors of ferroptosis may prevent the toxic reaction. Deferiprone, an orally bioavailable brain permeable, is one of the important iron chelators used in treatment of iron load. Its role in central nervous system has been studied especially in Parkinson’s disease. In a clinical randomized controlled trial, iron chelator deferiprone decreased iron accumulation in the substantia nigra which resulted in a slowing in the progression of motor dysfunction in Parkinson’s disease patients. Additionally, its effect was studied in other central nervous diseases as amyotrophic lateral sclerosis ^25^Yan et al., 2021 ; ^26^Klopstock et al., 2018; ^27^Moreau et al., 2018; ^28^Devos et al., 2014).

So, we aimed to determine if the cisplatin-induced nerve toxicity is intrinsically linked to changes in cellular iron homeostasis and related ferroptosis cell death pathways. We hypothesized that an iron chelator, deferiprone, might play a role in ameliorating peripheral neurotoxicity through hindering these cellular changes by using a deferiprone dose that enveloped the cisplatin dosage. This still a field that is not fully investigated in the literature.

## Materials and methods

### Animals

Twenty-four male Wistar rats were purchased from the National Research Centre animal house with an average weight of 200 g ± 20. All experimental procedures were approved by the animal ethics committee of Cairo University (approval no: CU III F 14 20). All rats were housed and maintained at a temperature (of 23 ± 2 °C) and under standard lighting conditions (12:12 h light-dark) with free access to water and a standard laboratory pellet diet.

### Experimental design

**Fig. 1:**
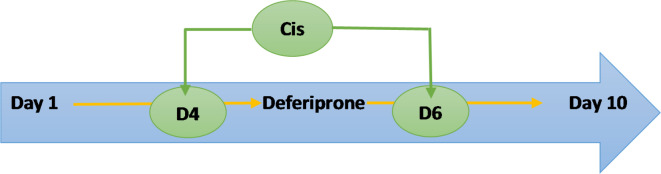
Method of drug administration.

Rats were randomly divided into four groups of six rats per group including the control group (Cont.), deferiprone group (Def), Cisplatin group (Cis) and Deferiprone + Cisplatin group (Cis + Def) (Fig. 1) in which:

**Group I: Cont. group**: the rats were treated with oral gavage of the vehicle (tween 80 and distilled water) of deferiprone for 10 days and injected with saline on days 4, 5, and 6 corresponding to Cisplatin injection.

**Group II: Def treated group**: the rats were treated by oral gavage of deferiprone for 10 days.

**Group III: Cis treated group**: single intraperitoneal (IP) injection of cisplatin on the 4th, 5th and 6th day of vehicle oral gavage for 10 days.

**Group IV: Cis + Def group**: single IP injection of cisplatin on the 4th, 5th and 6th day of Deferiprone 10 days administration by oral gavage.

At the end of the experimental period, all rats were subjected to physiological function assessment experiments. After that, they were anaesthetized with ketamine–xylazine in a dose of 100 mg /kg and 20 mg/kg, respectively, then sacrificed by cervical dislocation followed by sciatic nerve dissection for histopathological and biochemical analysis.

### Drug dosage

#### Cisplatin

Cisplatin (1 mg/ml sterile concentrate Mylan Co.) was given in a dose of 2 mg/Kg for 3 days (4th, 5th, and 6th days of the experimental period) (Fig. 1)^14,29,30^.

#### Deferiprone

Deferiprone (Ferriprox Apotex Inc.) a 500-mg, film-coated, IR tablet was given in 200 mg/Kg throughout the 10 days of the experiment pre and post-Cisplatin administration (Fig. 1)^23^.

### Physiological function assessment

#### Adhesive tape contact and removal test

This test was used to assess sensorimotor neurological function. A 3-day training session was conducted to ensure that the animal adapts to the condition of being put on the tape and to learn to tear the tape off within 10s. First, each rat was placed in a clear plexiglass box and allowed to habituate the experimental cage for 2 min. Then, gentle application of 5 mm × 5 mm sized adhesive tape to the ventral side of each forepaw in random order with average equal pressure. The rat was placed back into the box. The latency time of initial contact with either piece of tape and that of removing the tape using its teeth was recorded with a cutoff time around180s with an average of three trials with an interval of 5 min between each trial^31–34^.

#### Tail flick test

This test was used to evaluate pain threshold using heat painful stimulus in which tail latency time was measured in seconds from the initial heat source activation until tail withdrawal. Rats were gently held, and their tails were placed on the heat stimulus. The maximum cutoff was considered 30 s. Tail flick latency was measured twice at 30-minute intervals for each rat, and the average was calculated^35,36^.

#### Hot plate test

This test was used to assess sensitivity to thermal stimulation in which a radiant heat source delivering a heat stimulus was focused under the plantar surface of the hind paw. The hotplate (Ugo Basile Model 7280, Varese, Italy) was maintained at 52.0 ± 0.5 °C. The latency time from initiation of the stimulus until paw licking, lifting, or jumping was measured using a stopwatch. A cutoff time of 40 s was set so as to avoid tissue damage due to excessive heat. The test was repeated two times with a 30-minute difference between trials. The mean withdrawal latency was calculated^35,37,38^.

#### Cold allodynia test

This test was used to evaluate the cold pain sensitivity of rats by exposing the hind paw of each rat to ice-cold temperature (3–4 °C). The hind paw withdrawal latency time for each rat was determined. Only one hind paw was evaluated during each immersion at a time, with a cutoff time of 40 s. For each animal, two readings were taken for each hind paw at 5 min intervals, and the latency time was reported as the mean of both hind paws’ readings^35,36,39,40^.

#### Rotarod test

The balance and coordination of rats were assessed using an accelerating rotarod (Ugo Basile, Varese, Italy, Model 47750). All rats were trained for three consecutive days (three sessions for 5 min each). On the day of the experiment, rats were placed on a rotating rod in the direction that opposes the rotating rod (3 cm diameter and 90 cm height), starting at 4 rpm and accelerating linearly to 40 rpm. The performance of rats was assessed for a period of 5 min (300 s), and the latency time on the Rotarod was recorded for three consecutive test trials with an interval of 20–30 min. The latency to fall off the Rotarod was recorded^34,36,37,40–43^.

#### Nerve conduction velocity test

In vivo nerve conduction velocity (NCV) measurements using PowerLab 8SP system (AD Instruments, Australia) at a frequency of 10 Hz for recording and analyzing data while the stimulator used was Ugo Basil ECT Unit (Model 57800, Italy) was utilized to stimulate the right sciatic nerve with specific parameters: a pulse duration of 0.1 ms, an intensity of 25–50 mA, and a frequency of 50 Hz. The NCV recordings of the sciatic nerve, including motor and sensory nerve divisions, were performed in anesthetized rats using ketamine and xylazine in doses of 100 mg/Kg and 20 mg/kg, respectively. The body temperature of 37 °C was maintained automatically utilizing a heating pad. Sciatic nerves were stimulated at two sites using paired percutaneous needles connected to alligator clips electrodes; the electrodes were inserted at the sciatic notch and distal at the level of the knee joint with a surface electrode placed at the base of the tail to serve as a ground electrode. The motor action potential was measured in which the nerve is stimulated proximally, and the action potential was recorded distally. The nerve conduction velocity (m/s) is derived from the onset latency (ms) from stimulus to action potential. Measurements were taken 4 times, and the accepted result was their arithmetic average. Stimulation, recording, and ground electrodes were identical to standard “alligator” clips^35,41,44–47^.

### Biochemical assessment

#### Quantifying lipid peroxidation and cellular antioxidant content

After sacrifice, nerve tissues were dissected from animals, rinsed with phosphate-buffered saline (PBS) on ice. Then, they were gently blotted between the folds of a filter paper and weighed in an analytical balance. Using a polytron homogenizer, nerve tissues were homogenized at 4 °C in a 0.05 M PBS (pH 7.4). Centrifugation of the homogenate was undergone (10,000 rpm, 20 min.). The protein concentration in the supernatant (tissue homogenate) was measured using Lowry technique. Afterwords, the homogenate was separated and aliquoted. Based on the thiobarbituric acid reaction assay, a fresh homogenate aliquot was utilized for calculation of the amount of malondialdehyde (MDA) generated due to lipid peroxidation. Based on sulfhydryl group reducing activity, quantification of the reduced glutathione (GSH) content was performed. The rest of homogenate aliquots were processed for PCR analyses. All required reagents and kits were provided by Sigma-Aldrich.

#### Quantitative real-time PCR analysis

Following manufacturer’s manual, total RNA was extracted from freshly homogenized nerve tissues using the Direct-zol RNA Miniprep Plus Kit (CAT# R2072, Zymo Research Corp., United States). SuperScript IV One-Step RT-PCR kit (CAT# 12594100, Thermo Fisher Scientific, Waltham, MA, United States) was utilized to reverse transcribe extracted RNA into cDNA in accordance with the manufacturer’s instructions. The quantity and purity of the resulted cDNA were assessed using Q5000 UV-Vis Nanodrop (Quawell inc.). cDNA samples were aliquoted and stored at -80 °C until analyzed. SYBR Green PCR kit (Qiagen, Germany) was used to determine mRNA levels of cellular antioxidant-related genes; Glutathione peroxidase-4 (GPX4; CAT# QT01169434) and solute carrier family 7a member 11 (SLC7A11; CAT# QT00393841) as well as Ferritinophagy-related genes; Nuclear Receptor Coactivator 4 (NCOA4; CAT# QT01799378), Iron Responsive Element Binding Protein 2 (IREB; CAT# QT00177716) and Ferritin heavy chain 1 (FTH1; CAT# QT01817844). All primers used throughout the experiment were ready-to-use QuantiTect ^®^ Primer Assay kits purchased from Qiagen. Using the Rotor gene PCR system (Qiagen, Germany), three assays in duplicate of quantitative PCR was carried out at the following thermal profile cycling: 10 min at 95 °C (polymerase activation) followed by 40 cycles at 95 °C for 10 s, 58 °C for 15 s, and 72 °C for 15 s. After the PCR run, data were expressed in the cycle threshold (Ct) for each of the target gene and glyceraldehyde-3-phosphate dehydrogenase (GAPDH; CAT#QT00199633) housekeeping gene. Using the ΔΔCt method, normalization for variation in the expression of target genes was performed referring to the mean Ct expression values of the GAPDH. The relative quantitation (RQ) of each target gene is quantified according to the calculation of the 2^−ΔΔCt^ method.

### Histopathological and immunohistochemical assessment

#### Morphological study

Hematoxylin and Eosin (H & E) and Silver staining of neurofibrils^48^ were used for the histopathological study^49^. A tissue processor (Cat# 5903, SAKURA Finetek, Japan) was used to embed the nerves in paraffin after they had been submerged in a 10% formaldehyde solution for 24 h. The tissue samples were divided into slides that were 5 μm thick. The tissue samples were deparaffinized in xylene for three minutes twice before being rehydrated in a lowering concentration gradient of ethanol (100%, 90%, 80%, 70%, and 0% v/v in water) for hematoxylin and eosin (H&E) staining. After three minutes of incubation in hematoxylin solution (Cat# S3309, Dako, USA), the tissue samples were rinsed twice in distilled water for three minutes each to get rid of any remaining stain. They were then incubated for ten to fifteen seconds in the bluing reagent.

#### Immunohistochemical study

S100 Ab^50^: is mouse monoclonal IgG Ab (Thermo Fisher Scientific, USA catalog number: MA1-26621). It was supplied as 500 µl diluted 1:100. The reaction is cytoplasmic. The + ve control was melanoma and schwannoma.

Proliferating cell nuclear antigen (PCNA)^51^ (Clone PC10, Lab Vision Corporation, USA) immunostaining. Prediluted 1ry mouse monoclonal PCNA antibody was added 0.1 ml for 60 min. The reaction is nuclear. Human skin served as + ve control by omitting 1ry antibody addition for negative control preparation.

Morpho-quantitative Study: Leica Qwin 500 LTD (Cambridge UK) image analyzer, measurement of the count of myelinated, myelinating and unmyelinated nerve fibers, in addition to Schwann cells in H&E-stained sections in five non overlapping fields using interactive measurements menu. The area% of neurofibrils in silver-stained sections, the area of S100 + ve Schwann cells and that of PCNA + ve nuclei of Schwann cells, were performed by binary mode. All measurements done in the fields were selected from the specimens belonging to the four groups of rats in the prepared slides.

### Statistical analysis

Statistical analysis was performed using SPSS software (version 17.0; SPSS, United States). The parametric variable groups were compared using one-way ANOVA, followed by the Tukey post hoc test. The Kruskal–Wallis test compared the groups of nonparametric variables. Graphs were sketched using GraphPad Prism (United States) version 7 software. The values of *p* < 0.05 were considered to indicate a statistically significant difference.

## Results

### Deferiprone embracing cisplatin dosage improves nerve physiological functions affected by Cis-induced polyneuropathy

#### Adhesive tape test to assess the sensorimotor neurologic deficits of both forepaws by using bilateral tactile stimulation

##### Adhesive tape contact test (AT-CT)

**Fig. 2:**
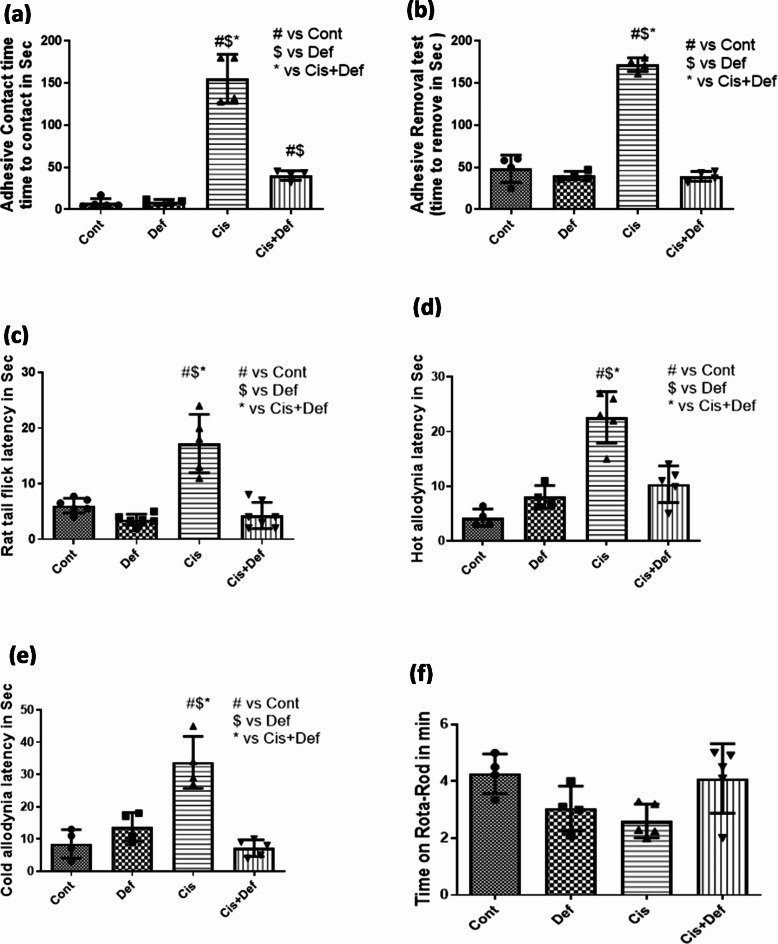
(**a**–**f**): showed the statistical results of the physiological functional assessment of different sensory and motor nerves including the tail, upper and lower limbs regarding adhesive tape contact test (**a**), adhesive tape removal test (**b**), tail flick test (**c**), hot allodynia test (**d**), cold allodynia test (**e**) and Rotarod test (**f**). # vs. control $ vs. Def *vs Cis P value ≤ 0.05. All the data was recorded as mean ± SE.

The Cis-treated rats showed significant deterioration in the time needed for adhesive tap contact compared with the control group (*P* < 0.0001) while the Cis + Def treated group showed significant improvement compared by the Cis group (*P* < 0.0001). However, the Cis + Def group was still showing significant prolongation compared to the Cont. group (*P* < 0.05). This indicated improvement in the group treated by Def starting before Cis administration (Fig. 2a and supplementary Table 1).

##### Adhesive tape removal test

The Cis-treated rats showed significant prolongation in time needed for adhesive tap removal compared with the control group (*P* < 0.0001) while the Cis + Def treated group showed significant improvement compared to the Cis group (*P* < 0.0001) reaching non-significant difference compared to the Cont. group (*P* > 0.05). This indicated improvement in the group treated by Def starting before Cis administration (Fig. 2b and supplementary Table 1).

#### Tail flick test

The tail flick test measures the withdrawal reflex arc in relation to painful thermal stimulation. The Cis treated rats showed significant prolongation in time needed for tail flick due to hot painful stimulation compared with the control group (*P* < 0.0025), while the Cis + Def treated group showed significant improvement compared to the Cis group (*P* < 0.0001) reaching non-significant difference compared to the Cont. group (*P* > 0.05). This indicated improvement in the group treated by Def starting before Cis administration (Fig. 2c and supplementary Table 1).

#### Hot plate test

The hot plate test measures latency to hot temperature, affecting the withdrawal reflex arc in lower limbs. The Cis treated rats showed significant latency for the start of jumping due to hot temperature stimulation compared with the control group (*P* < 0.025) while the Cis + Def treated group showed significant improvement compared by the Cis group (*P* < 0.05) reaching a non-significant difference compared to the Cont. group (*P* > 0.05). This indicated improvement in the group treated by Def starting before Cis administration (Fig. 2d and supplementary Table 1).

#### Cold allodynia test

Cold allodynia test measures the latency to cold temperature of paw withdrawal against cold temperature. The Cis-treated rats showed significant latency for the start of jumping due to cold temperature stimulation compared with the control group (*P* < 0.0001) while the Cis + Def treated group showed significant improvement compared to the Cis group (*P* < 0.0001) reaching a non-significant difference compared to the Cont. group (*P* > 0.05). This indicated improvement in the group treated by Def starting before Cis administration (Fig. 2e and supplementary Table 1).

#### Rotarod test

The Rotarod test measures latent time rotating and detects proprioception sensation as part of balance and motor coordination. There were no significant differences between groups. However, Cis-treated rats showed decreased latency for staying on Rotarod compared to the control group while the Cis + Def treated group showed prolonged stay on rotarod compared to the Cis group. (Fig. 2f and supplementary Table 1).

### Cisplatin dosage encompassed with deferiprone improved nerve conduction velocity (NCV) of sensory and motor nerves

#### Sciatic nerve motor division NCV

**Fig. 3:**
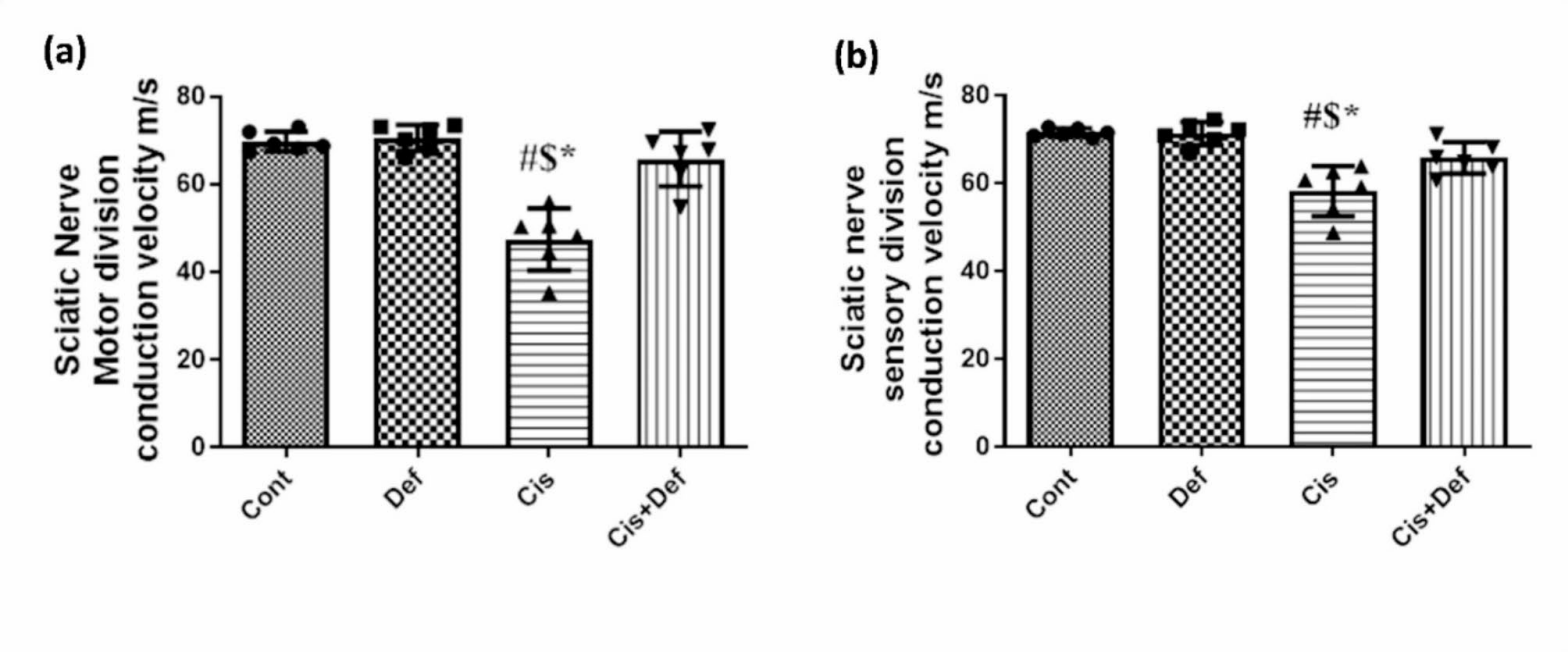
(**a–b**): (**a**) showed the result of nerve conduction velocity of the Motor division of the Sciatic nerve. (**b**) showed the result of nerve conduction velocity of sensory division of the Sciatic nerve. # vs. control $ vs. Def *vs Cis + Def P value ≤ 0.05. All the data was recorded as mean ± SE.

The Cis-treated rats showed significant deterioration compared with the control group (*P* < 0.0001) while the Cis + Def treated group showed significant improvement compared to the Cis group (*P* < 0.0001), reaching a non-significant difference compared to the Cont. group (*P* > 0.05). This indicated improvement in the group treated by Def starting before Cis administration (Fig. 3a and supplementary Table 1).

#### Sciatic nerve sensory division NCV

The Cis treated rats showed significant deterioration compared with the control group (*P* < 0.0001) while the Cis + Def treated group showed significant improvement compared to the Cis group (*P* < 0.0092), reaching a non-significant difference compared to the Cont. group (*P* > 0.05). This indicated improvement in the group treated by Def starting before Cis administration (Fig. 3b and supplementary Table 1).

### Iron chelation reduced iron-dependent lipid peroxidation that has been detected as a hallmark of ferroptosis and improved the glutathione antioxidant system, the main regulator counteracting ferroptosis

#### The effect on the nerve tissue biochemical level of Malondialdehyde (MDA) as a measurement for lipid peroxidation

**Fig. 4:**
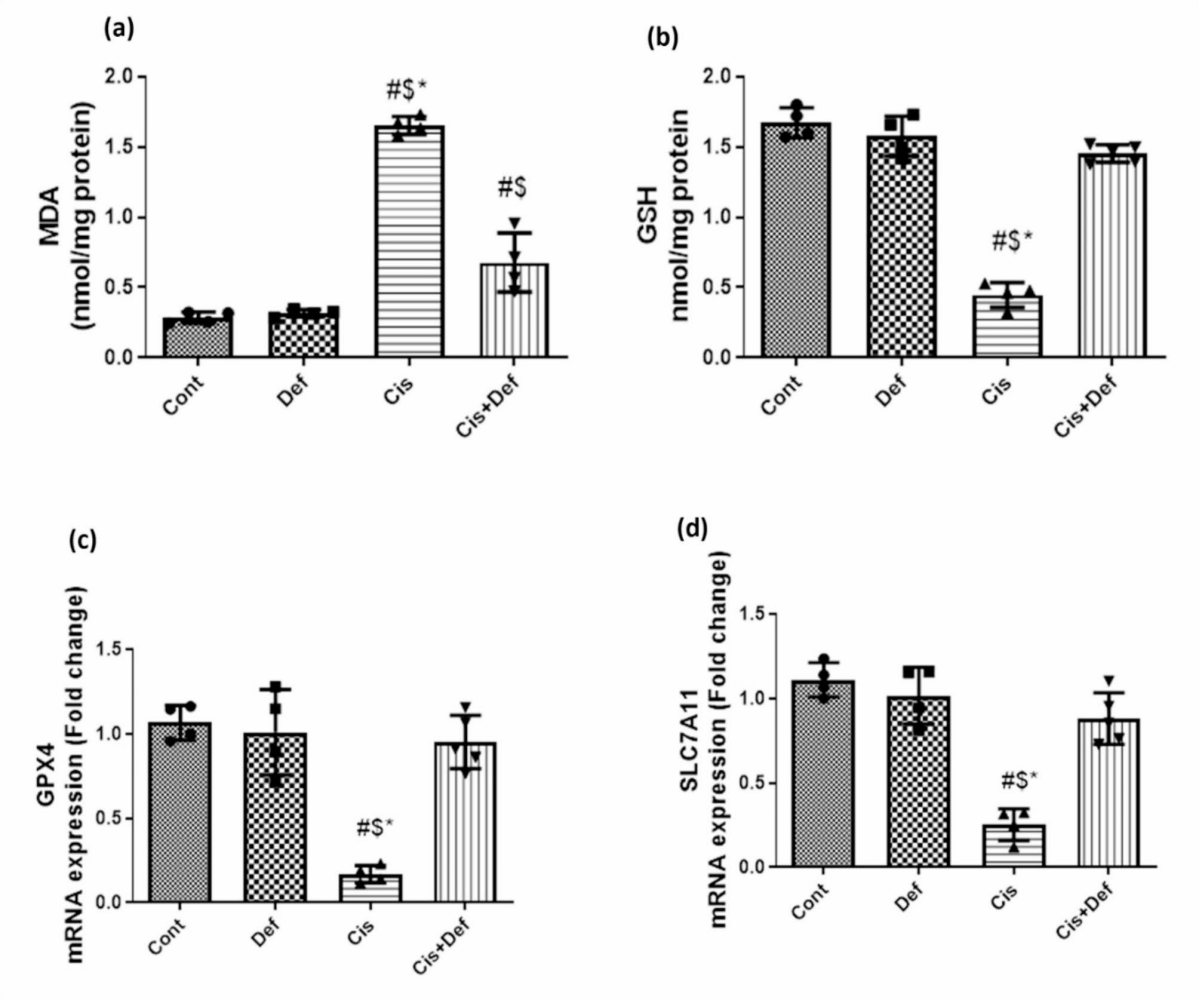
(**a** - **d**): Effect of Deferiprone (Def) treatment (200 mg/Kg) on cellular oxidative stress/antioxidant system. (**a** & **b**) Biomarkers for lipid peroxidation (malondialdehyde; MDA) and antioxidant content (glutathione, GSH) respectively. Data are represented as the mean ± SE. (**c** & **d**) mRNA expression level of cellular antioxidant-related genes; GPX4 and SLC7A11 respectively. mRNA expression data are recorded as the mean ± SE of three assays in duplicate normalized to GAPDH and represented as fold changes as compared with the mRNA levels of the control group. # vs. control $ vs. Def *vs Cis + Def, P value ≤ 0.05.

The Cis-treated rats showed significant increase in lipid peroxidation compared with the control group (*P* < 0.0001) while the Cis + Def treated group showed significant decrease compared to the Cis group (*P* < 0.0001). However, the Cis + Def group showed a significant increase compared to the Cont. group (*P* < 0.025). This indicated improvement in the group treated by Def starting before Cis administration in improving peroxidative stress (Fig. 4a and supplementary Table 2).

#### The effect on the nerve tissue biochemical level of the most potent antioxidant glutathione (GSH)

The Cis-treated rats showed a significant decrease in GSH level compared with the control group (*P* < 0.0001), while the Cis + Def treated group showed significant increase compared to the Cis group (*P* < 0.0001). The improvement in the level of GSH showed by the Cis + Def group reached the Cont. group level (*P* > 0.05). This indicated the prophylactic effect of Def dosage enclosing Cis one in regulating the antioxidant system (Fig. 4b and supplementary Table 2).

#### The effect on glutathione peroxidase 4 (GPX4) expression in nerve tissue which is the vital (key) regulator of ferroptosis inhibition

The Cis-treated rats showed significant down regulation in the expression of mRNA of GPX4 assessed by quantitative RT-PCR compared with the control group (*P* < 0.0001) while the Cis + Def treated group showed significant upregulation compared to the Cis group (*P* < 0.0001). The improvement in GPX4 expression showed by the Cis + Def group reached the Cont. group expression with no significant difference (*P* > 0.05). This emphasized the prophylactic effect of Def dosage rapping Cis one regarding adjusting the antioxidant system (Fig. 4c and supplementary Table 2).

#### The effect on solute carrier family 7a member 11 (SLC7A11) expression in nerve tissue, which is the upstream of oxidative and lipid peroxidative stresses and so is considered a ferritinophagy inhibitor

The Cis-treated rats showed significant down regulation in the expression of mRNA of SLC7A11 assessed by quantitative RT-PCR compared with the control group (*P* < 0.0001) while the Cis + Def treated group showed significant upregulation compared by Cis group (*P* < 0.0001). The improvement in SLC7A11 expression showed by the Cis + Def group reached the Cont. group expression with no significant difference (*P* > 0.05). This underlined the protective effect of Def dosage rapping Cis one in improving antioxidant system and inhibiting ferritinophagy (Fig. 4d and supplementary Table 2).

### Rapping the cisplatin dosage with pre/post administration of deferiprone impacts iron homeostasis by impacting ferritinophagy at tissue level

#### The effect on nuclear receptor coactivator 4 (NCOA4) in nerve tissue which is one of the ferritinophagy inducing genes

**Fig. 5:**
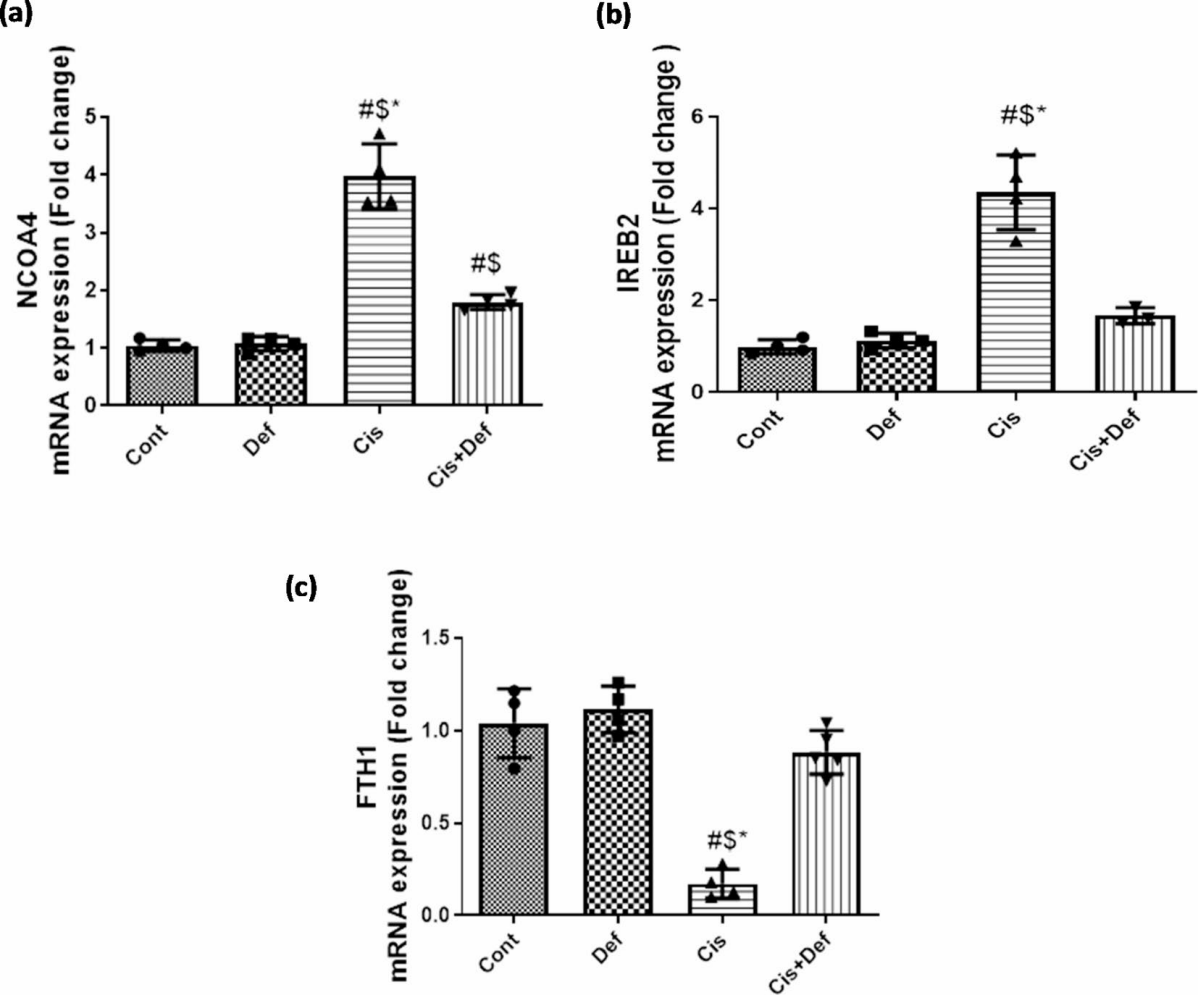
(**a** - **c**): Effect of Deferiprone (Def) treatment (200 mg/Kg) on mRNA expression level of Ferritinophagy-related genes following Cisplatin-induced neuronal toxicity (**a**) NCOA4 (**b**) IREB (**c**) FTH1. mRNA expression data are recorded as the mean ± SE of three assays in duplicate normalized to GAPDH and represented as fold changes as compared with the mRNA levels of the control group. # vs. control $ vs. Def *vs Cis + Def, P value ≤ 0.05.

The Cis-treated rats showed a significant upregulation in the expression of mRNA of NCOA4 assessed by quantitative RT-PCR compared with the control group (*P* < 0.0001) while the Cis + Def treated group showed significant down regulation compared to the Cis group (*P* < 0.0001). However, the down regulation of NCOA4 expression showed by the Cis + Def group did not reach the Cont. group expression with significant difference (*P* < 0.05). This highlighted the protective role of Def dosage rapping Cis one in reducing ferritinophagy (Fig. 5a and supplementary Table 2).

#### The effect on iron responsive element binding protein 2 (IREB2) in nerve tissue which is one of the ferritinophagy inducing genes

The Cis-treated rats showed significant upregulation in the expression of mRNA of IREB2 assessed by quantitative RT-PCR compared with the control group (*P* < 0.0001) while th Cis + Def treated group showed a significant down regulation compared to the Cis group (*P* < 0.0001) reaching the Cont. group expression values with no significant difference (*P* > 0.05). This emphasized the protective role of Def dosage rapping Cis one in reducing ferritinophagy (Fig. 5b and supplementary Table 2).

#### The effect on Ferritin heavy chain 1 (FTH1) in nerve tissue, which is one of the ferritinophagy inhibiting genes

The Cis-treated rats showed significant down regulation in the expression of mRNA of FTH1 assessed by quantitative RT-PCR compared with the control group (*P* < 0.0001) while the Cis + Def treated group showed significant upregulation compared to the Cis group (*P* < 0.0001) reaching the Cont. group expression values with no significant difference (*P* > 0.05). This finetuned the protective role of Def dosage rapping Cis one in reducing ferritinophagy (Fig. 5c and supplementary Table 2).

### Histopathological and immunohistological tissue assessment

#### Hematoxylin and eosin (H&E) histopathological results

**Fig. 6:**
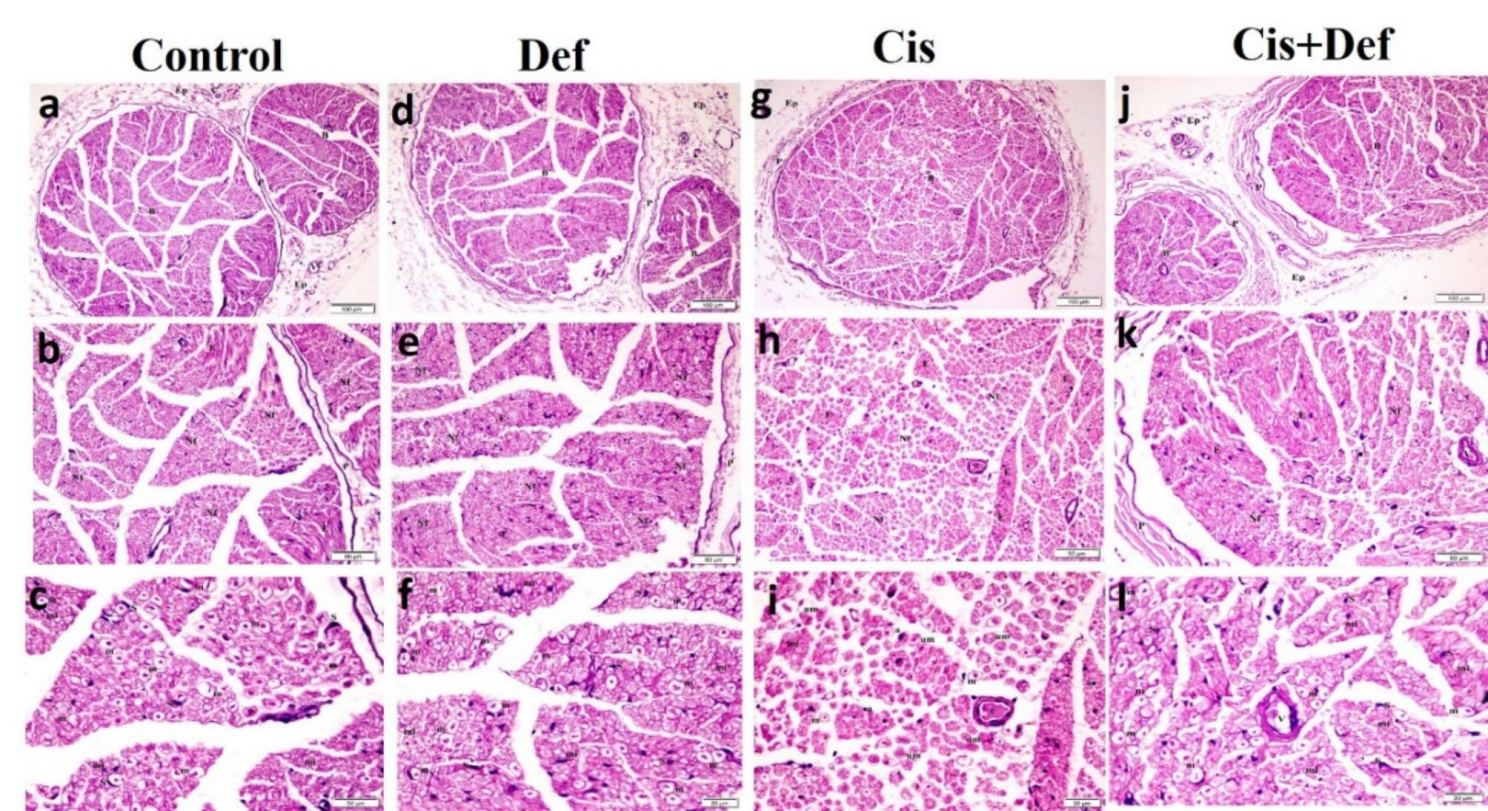
(**a–l**): Sections in the sciatic nerve of rats (H&E) showing: (**a&d**): apparently normal bundles (B) of nerve fibers surrounded by perineurium (P) and covered by epineurium (Ep) containing arterioles (**a**) and venules (V) (x 100), (**b&e**): multiple nerve fibers (Nf), endoneurium (E) and perineurium (P) (x 200), (**c&f**): multiple myelinated (m), some myelinating (mi) nerve fibers and some Schwann cells (S) (x 400) in control and Def groups. In cis group: (**g**): apparently abnormal bundles (B) of nerve fibers surrounded by perineurium (P) and covered by epineurium (Ep) containing a congested vessel (c) (x 100), (**h**): multiple atypical nerve fibers (Nf), endoneurium (E), perineurium (P) and a congested vessel (c) (x200), (**i**): apoptotic Schwann cells (a), a congested vessel (c), multiple unmyelinated (um), some myelinating (mi) and few myelinated (m) nerve fibers (x400). In combined Cis + Def group: (**j**): apparently normal bundles (B) of nerve fibers surrounded by perineurium (P) and covered by epineurium (Ep) containing arterioles (a) and venules (V) (x 100), (**k**): multiple nerve fibers (Nf), endoneurium (E), perineurium (P) and a venule (V) (x 200), (**I**): multiple myelinated (m), multiple myelinating (mi) nerve fibers and multiple Schwann cells (S) (x400).

H&E - stained sciatic nerve sections of different groups. Cont. and Def groups (Fig. 6a–f) showed apparently normal bundles of nerve fibers surrounded by perineurium and covered by epineurium containing arterioles and venules (Fig. 6b&e), multiple nerve fibers, endoneurium and perineurium (Fig. 6c&f), multiple myelinated, some myelinating nerve fibers and some Schwann cells were seen. In the Cis group (Fig. 6g - i), apparently abnormal bundles of nerve fibers surrounded by perineurium and covered by epineurium containing congested vessels (Fig. 6g) were observed. In addition to multiple atypical nerve fibers, endoneurium, perineurium and congested vessels (Fig. 6h). Apoptotic Schwann cells, congested vessels, multiple unmyelinated, some myelinating and a few myelinated nerve fibers were also detected (Fig. 6i). Moreover, in the Cis + Def group (Fig. 6j–l), apparently normal bundles of nerve fibers surrounded by perineurium and covered by epineurium containing arterioles and venules (Fig. 6j). Multiple nerve fibers, endoneurium, perineurium and venules (Fig. 6k) in addition to Multiple myelinated, multiple myelinating nerve fibers and multiple Schwann cells were seen (Fig. 6l).

#### Immunohistochemical results

**Fig. 7:**
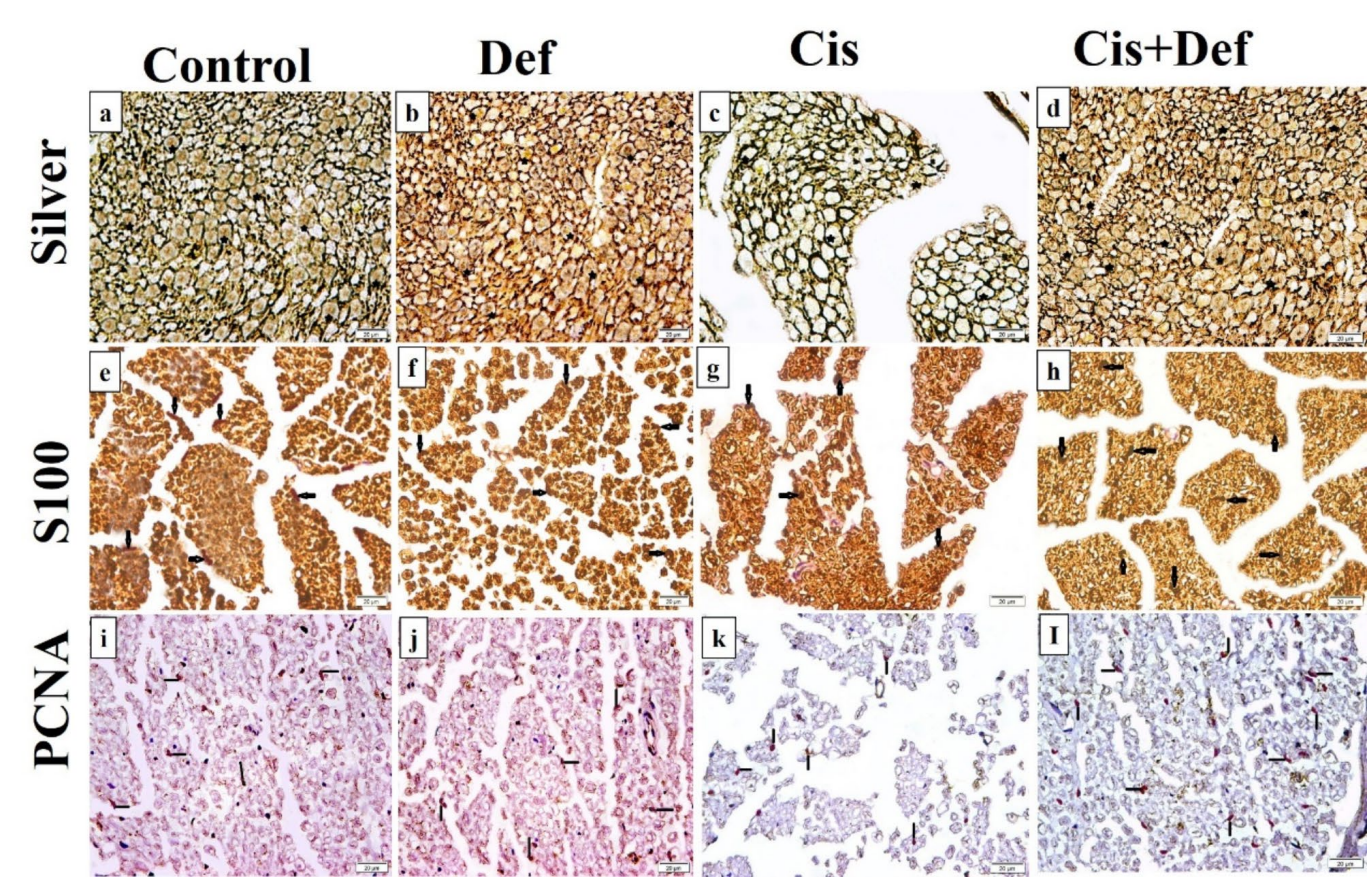
(**a**–**l**): Sections in the sciatic nerve of rats (x400) with immunostaining in which (**a**–**d**) showed Silver-staining in which (**a** & **b**): neurofibrils (stars) in numerous nerve fibers in the Cont and Def groups respectively. (**c**): neurofibrils (stars) in few nerve fibers in cis group, (**d**): neurofibrils (stars) in multiple nerve fibers in the Cis + Def group. Additionally, (**e**–**h**) showed S100 immunostaining in which (**e** & **f**): some + ve Schwann cells (arrows) in the Cont and Def groups respectively, (**g**): a few + ve Schwann cells (arrows) in the Cis group, (**h**): multiple + ve Schwann cells (arrows) in the Cis + Def group. Finally, (**i**–**l**) showed PCNA immunostaining in which (**i** & **j**): some + ve Schwann cells nuclei (lines) in the control and Def groups respectively, (**k**): few + ve Schwann cells nuclei (lines) in the Cis group, (**l**): multiple + ve Schwann cells nuclei (lines) in the Cis + Def group.

Silver-stained sections in the sciatic nerve of rats of different groups (Fig. 7a–d) showed neurofibrils in numerous nerve fibers in the Cont. and Def groups (Fig. 7a & b, respectively). Neurofibrils in a few nerve fibers in the Cis group were seen (Fig. 7c). In group IV (Cis + Def), neurofibrils in multiple nerve fibers were also detected (Fig. 7d).

S100 immunostaining sections (Fig. 7e–h) demonstrated some + ve Schwann cells in the Cont and Def groups (Fig. 7e & f, respectively), few + ve Schwann cells in the Cis group (Fig. 7g) and multiple + ve Schwann cells (Fig. 7h) in the Cis + Def group.

Proliferating cell nuclear antigen (PCNA) immunostaining (Fig. 7 i–l) showed some + ve Schwann cells nuclei in the Cont and Def groups (Fig. 7i&j respectively), while few + ve Schwann cells nuclei in the Cis group (Fig. 7k) and multiple + ve Schwann cells nuclei in the Cis + def group (Fig. 7l).

#### Morphometric results

**Fig. 8:**
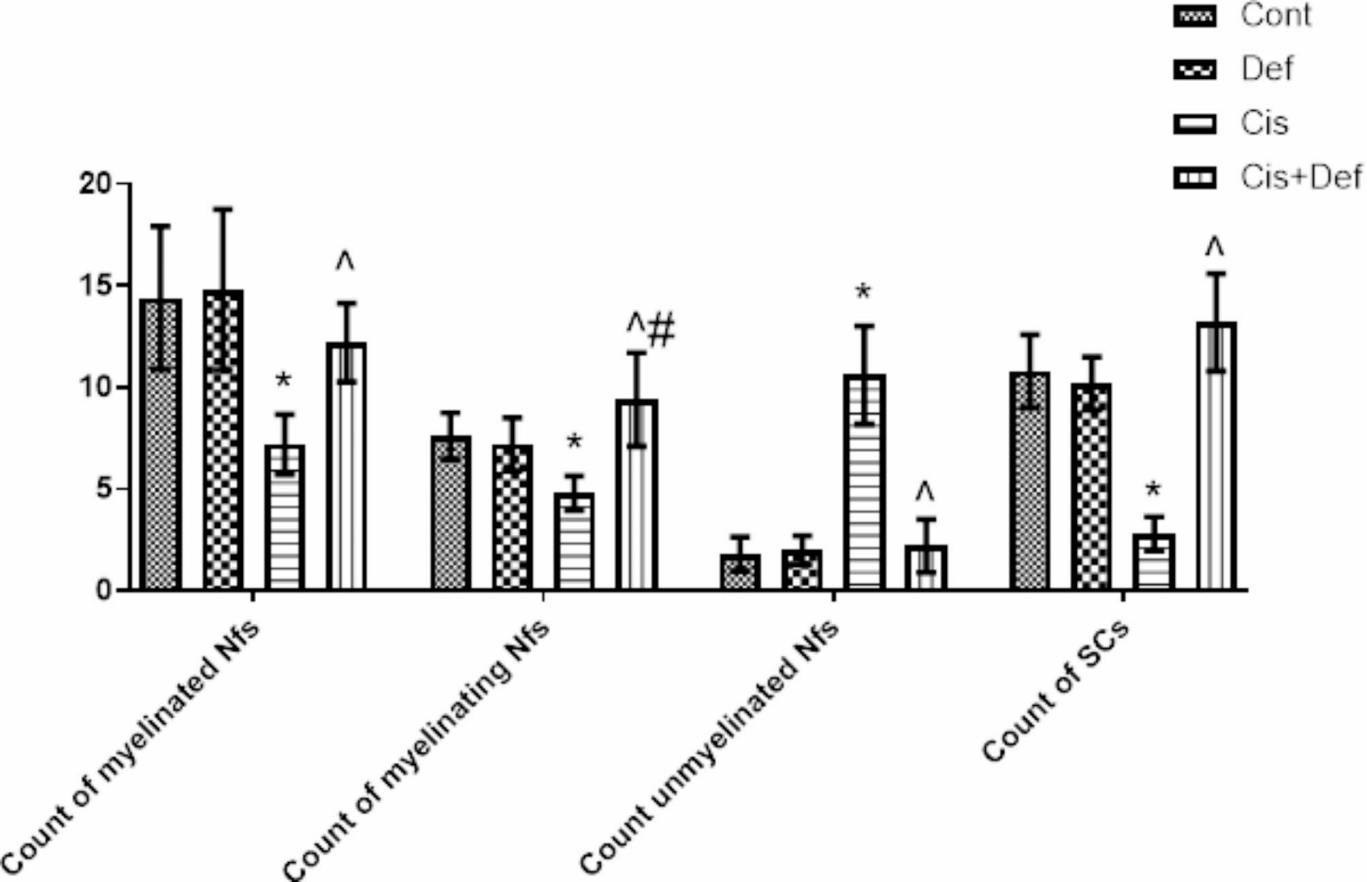
Shows a histogram illustrating mean count of myelinated, myelinating, unmyelinated nerve fibers and Schwann cells in which the Series: (1) Myelinated (2) Myelinating (3) Unmyelinated nerve fibers (4) Schwann cells. Group I: Cont., Group II: Def, Group III: Cis, Group IV: Cis + Def. *P* ≤ 0.05 *increase/decrease versus other groups, ^ increase/decrease versus cisplatin group, # increase versus other groups.

In the Cis group, the mean count of myelinated, actively myelinating and unmyelinated nerve fibers, in addition to the count of Schwann cells (SCs) proved a significant decrease, except for the count of unmyelinated nerve fibers versus the other groups. On the other hand, in the Cis + Def group a significant increase was recorded in mean count of myelinated fibers versus Cis group. A significant increase was found in mean count of myelinating fibers in the Cis + Def group versus the other groups which indicated the positive impact of embracing dosage of Def to Cis one being started before and continuing after enclosing the whole period of Cis side effect (Table 1; Fig. 8).

**Fig. 9:**
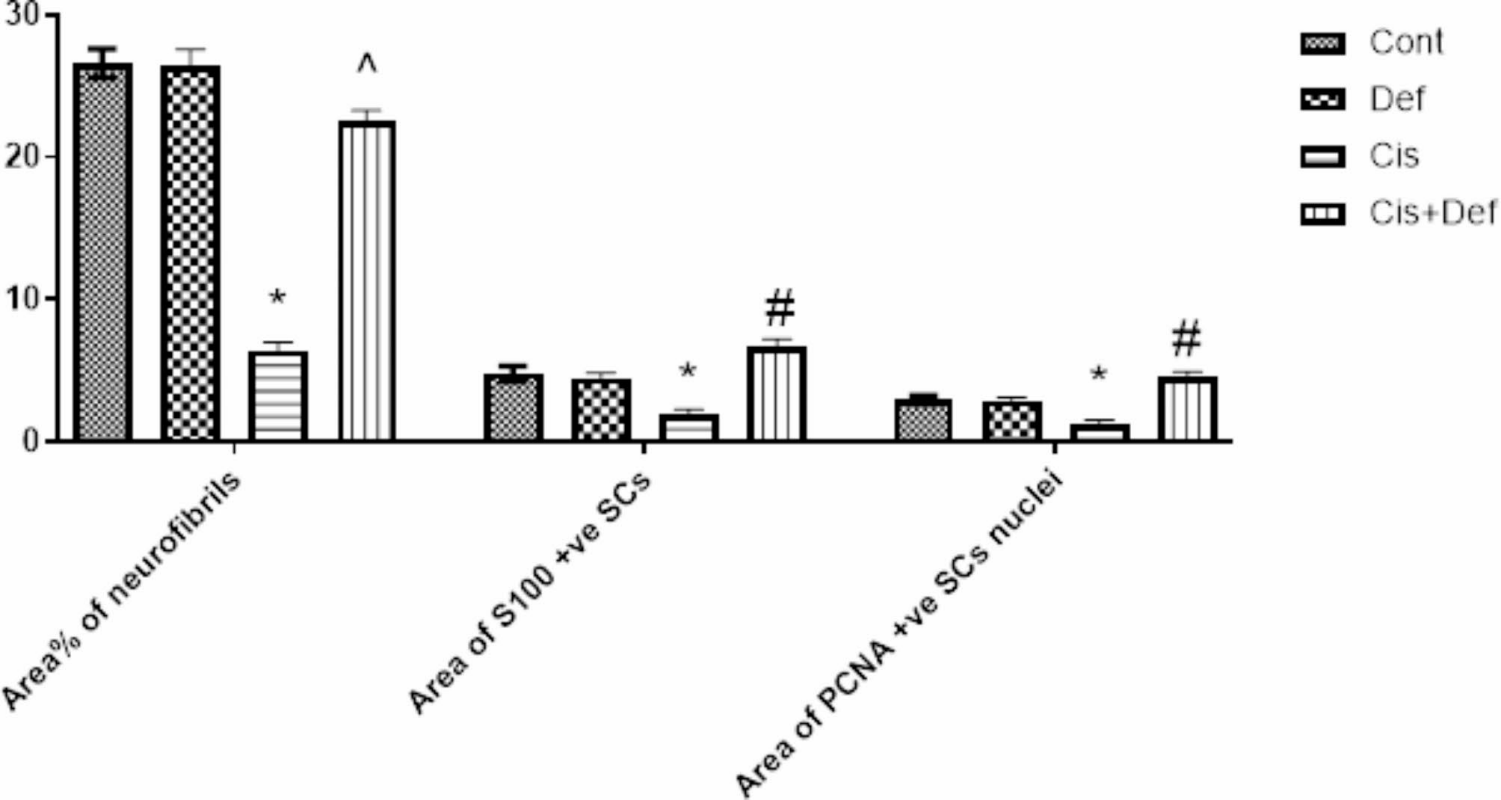
Shows a histogram of mean area% of neurofibrils, area of S100 + ve SCs, and area of PCNA + ve SCs nuclei. Series: (1) Area% neurofibrils (2) Area S100 + ve SCs (3) Area PCNA + ve SCs nuclei. SCs: Shwan cells. Group I: Cont., Group II: Def, Group III: Cis, Group IV: Cis + Def. *P* ≤ 0.05 *decrease versus other groups, ^ increase versus cisplatin group, # increase versus other groups.

In the Cis group, the mean area % of neurofibrils, mean area of S100 + ve SCs and mean area of PCNA + ve SCs nuclei proved a significant decrease versus the other groups. On the other hand, in the Cis + Def group a significant increase was recorded in mean area% of neurofibrils versus the Cis group. A significant increase was found in mean area of S100 + ve SCs and that of PCNA + ve SCs nuclei in the Cis + Def group versus the other groups which indicated improving activity of these cells highlighting the positive effect of Def dosage that started before and continued after Cis dosage (Table 2; Fig. 9).

**Table 1:** Mean ± SD count of myelinated, myelinating, unmyelinated nerve fibers (nfs) and Schwann cells (SCs).

Groups	Count of myelinated Nfs	Count of myelinating Nfs	Count of unmyelinated Nfs	Count of SCs
I. Cont group	14.5 ± 2.23	7.6 ± 1.51	1.8 ± 0.05	10.8 ± 1.05
II. Def group	14.8 ± 2.40	7.2 ± 1.39	2 ± 0.04	10.2 ± 1.02
III. Cis group	7.2 ± 1.43*	4.8 ± 0.92*	10.6 ± 1.55*	2.8 ± 0.33*
IV. Cis + Def group	12.2 ± 2.65^	9.4 ± 1.61^#	2.2 ± 0.17^	13.2 ± 1.52^

*P* ≤ 0.05 *increase/decrease versus other groups, ^ increase/decrease versus cisplatin group, # increase versus other groups.

**Table 2:** Mean ± SD area% of neurofibrils, area of S100 + ve SCs, and area of PCNA + ve SCs nuclei.

Groups	Area% of neurofibrils	Area of S100 + ve SCs	Area of PCNA + ve SCs nuclei
I. Cont group	26.4 ± 3.03	4.78 ± 0.52	2.96 ± 0.08
II. Def group	26.42 ± 2.91	4.48 ± 0.41	2.92 ± 0.07
III. Cis group	6.4 ± 1.32*	1.93 ± 0.51*	1.22 ± 0.25*
IV. Cis + Def group	22.54 ± 3.65^	6.72 ± 1.09#	4.52 ± 0.47#

*P* ≤ 0.05 *decrease versus other groups, ^ increase versus cisplatin group, # increase versus other groups.

## Discussion

This study investigated the potential neuroprotective effects of deferiprone, an iron chelator, against cisplatin-induced polyneuropathy and explored the underlying mechanisms involving ferritinophagy modulation. The cisplatin-induced neurotoxicity was evidenced by impaired sensory and motor nerve functions in various peripheral nerves, as indicated by prolonged withdrawal reflexes in hot plate, cold allodynia, and tail flick tests. The observed decrease in nerve conduction velocity (NCV) in both sensory and motor fibers of the sciatic further corroborate the neurotoxic effects of cisplatin. Histopathological examination revealed cisplatin-induced damage to nerve fibers, including demyelination and Schwann cell apoptosis. Our findings demonstrate significant improvements in nerve function, histopathology, and molecular markers of ferritinophagy when cisplatin treatment was combined by deferiprone administration.

Cisplatin, despite being important chemotherapeutic, displays a range of severe side effects due to its poor selectivity for cancerous tissue over normal tissue. Adding to that, the resistance of tumors demands high drug dosage which may aggravate side effects. One of the ugly faces is neurotoxicity which may interfere with daily life activity, affecting socioeconomic outcomes. Despite, the well-defined Cisplatin-induced oxidative stress has been considered the basis of neurotoxicity^30,52^, antioxidants did not give a final solution for that side effect. So, there is another hidden player behind the scene. One of the Cisplatin induced pathways to combat tumors is ferritinophagy which may end up in ferroptosis. So, we aimed to investigate the role of ferritinophagy in cis-induced neuropathy and hypothesized that the use of iron chelator dosage might ameliorate the neurotoxicity induced by Cisplatin due to ferritinophagy induction in nerve tissue.

In the current study using Deferiprone, as an iron chelator, embracing Cisplatin dosage improved Cis-induced polyneuropathy at the histopathological and physiological functional levels. This improvement was through the ferritinophagy pathway adjustment in which the ferritinophagy inhibitors including GSH, GPX4, SLC7A11 and FTH1 were upregulated while the ferritinophagy inducers including MDA, IREB1 and NCOA4 were downregulated. This was performed and discussed in the following detailed steps.

First, the neurotoxicity of cisplatin was assessed using multiple tests to confirm neuropathy of different nerves and neuronal types. The used protocol showed significant deteriorated sensory and motor nerve function. Prolonged withdrawal reflex was detected in hot plate testing cold allodynia, and tail flick tests indicated hypoalgesia to pain sensation in different nerve fibers^38,53,54^. In addition, adhesive tape contact and removal showed delayed response. It assessed the sensorimotor neurologic deficits of both forepaws by using bilateral tactile stimulation. The behavior included correct paw and mouth sensitivity (time-to-contact) and correct dexterity (time-to-remove)^32,33^. However, Seto and his colleagues showed mechanical allodynia but they used different dose of cisplatin (4 mg/Kg once weekly for two weeks)^38^. It has been shown that cisplatin accumulates especially in the dorsal root ganglia and causes nucleolar damage together with Schwan cells^55^. It has been known that cisplatin could decrease the circulating levels of nerve growth factor which plays potent trophic effect on the dorsal root ganglion neurons^12^. The nerve tissue damage was mainly sensory, while motor neuropathy could be also manifested^53,56^.

Moreover, NCV was significantly decreased at the level of the sensory and motor nerve fibers in the sciatic nerve. The sciatic nerve, the largest nerve of the peripheral nervous system, contains both large and small axons of the motor and sensory system^46^. Boehmerle and his colleagues also showed a significant decrease in the caudal sensory nerve action potential amplitude and a moderate reduction of nerve conduction velocity for cisplatin^41^. Since the NCV was highly dependent on rapid signal propagation augmented by myelination, demyelinating processes generally showed decreased conduction velocities^46^. As well Akman group showed that electromyographic findings revealed that compound muscle action potential amplitude was significantly decreased and distal latency was prolonged in the nontreated cisplatin-injected rats compared with the control group^56^.

On the other hand, the Rotarod test which detects position sense, coordination and equilibrium showed decreased latency, was insignificant. In line with this study, Boehmerle and his colleagues detected that behavioral testing revealed normal motor coordination^41^. However, Abdelsameea and Kabil showed significant deterioration in rotarod results but, they used different dosage 2 mg/kg intraperitoneal, twice weekly for five consecutive weeks^53^. This could be explained by the fact that proprioceptive sensation is also helped by vestibular and visual inputs as vestibular-ocular reflex that might have masked any deterioration in the sensation^57^. In addition, the effect of the given dosage in our experiment might have not affected the cerebellum that might need higher dosage^11,58^.

Secondly, the nerve tissue damage was confirmed by histopathological examination that showed apparently abnormal bundles of nerve fibers surrounded by perineurium and covered by epineurium containing congested vessels. In addition, there were multiple atypical nerve fibers, endoneurium, perineurium and apoptotic Schwann cells, multiple unmyelinated, some myelinating, and few myelinated nerve fibers^41,58^. Moreover, sliver immunostaining concluded the presence of neurofibrils in a few nerve fibers with a few Schwann cells that was confirmed by PNCA nucleoli immunostaining. The mean count of myelinated, actively myelinating and unmyelinated nerve fibers, in addition to the count of Schwann cells proved to be significantly decreased, except for the count of unmyelinated nerve fibers versus the other groups. The mean area% of neurofibrils, mean area of Silver and PCNA stained Schwan cells nuclei also significantly decreased compared to the other groups. This emphasized the demyelinating toxic effect of cisplatin together with its DNA damaging effect on Schwan cells^59,60^. Cisplatin affects Schwann cells, which have an important role in the nerve development and regeneration^55^. The accumulated reactive oxygen species might have been the trigger for axonal degeneration and interruption of its transportation^56,61^. On the other hand, Abdelsameea and Kabil showed atrophy and fragmentation of the nerve fibers with decreased expression of myelin basic protein in the myelin sheath in immune-stained sections. However, they used another dosage mechanism as stated previously^53^.

Thirdly, the effect of Cisplatin inducing ferritinophagy in neuronal tissue was confirmed by detecting its inducers and suppressors together with ferroptosis tendency. These were measured by the lipid peroxidation and deterioration of the antioxidant family. Nerve tissue level of Malondialdehyde (MDA) as a measurement for iron-dependent lipid peroxidation was significantly elevated^7,30,62,63^. Together with the main antioxidant family represented by GSH, GPX4 and SLC7A11 were significantly decreased and down regulated respectively^30,64^. Ferroptosis, a newly identified type of regulated cell death, has been affected by lipid peroxidation and reactive oxygen species accumulation which have been considered good evidence^65^. Solute carrier family 7 member11 (SLC7A11) plays a critical role in the negative regulation of ferroptosis by preventing GSH depletion^66^. Cisplatin is considered a ferroptosis inducer^8^. Akman and his group showed elevated level of plasma MDA and decreased level of GSH with cisplatin induced neuropathy^53,56,67^. Liang and his colleagues showed that an analog of erastin augmented cisplatin effects against non-small cell lung cancer cells by inducing ferroptosis detected by upregulation of ROS, and lipid peroxidation in addition to downregulated GPX4, the key regulator of ferroptosis inhibition^7,52^. The loss of the glutathione peroxidase GPX4 activity that is considered lipid repair enzyme could trigger ferroptosis accumulation of lipid-based reactive oxygen species^25,50,63,68,69^. GPX4 has distinct activity to prevent uncontrolled peroxidation of phospholipids. It is considered the most central downstream ferroptosis regulator^7,50,70,71^. So, this suggested that ferritinophagy was exaggerated ended up with ferroptosis. As ferritinophagy is vital to disturb the cellular iron equilibrium and empowering the production of oxygen radicals in perinuclear compartments during ferroptosis^72,73^.

The upregulation of ferritinophagy inducing genes: NCOA4, IREB2, and down regulation of FTH1 by cisplatin in nerve tissue has been confirmed in the present study. NCOA4 is the cargo receptor autophagic/lysosomal degradation of ferritin liberating iron intracellular. NCOA4-mediated ferritinophagy plays an important role in the initiation of ferroptosis^72,74–76^. IREB2 regulates iron homeostasis by altering the iron-regulatory proteins /iron-responsive element (IRE) ratio to further increase intracellular free iron and is closely related to ferroptosis induction^72,77^ FTH1 is considered down regulator gene and one of the critical mediators in the ferroptosis procedure and major iron storage protein in which its down regulation indicated ferritinophagy^66,78,79^.

Despite the Cisplatin oxidative stress and proven relationship to neuropathy and the use of antioxidants in multiple studies, there was still manifested side effect on neuronal tissue which pointed to a masked player that may affect the prognosis^30,53,54,64,67,80–86^. The question is, is there something that lies behind the oxidative stress? May be the role of iron and its impact in ferritinophagy / ferroptosis pathway. Ferroptosis plays important role as an anti-tumor pathway, however the activation of ferroptosis and dysfunctional ferritinophagy could accelerate neurodegeneration diseases^72^. In addition, iron is an essential part of driving intracellular lipid peroxidation and ferroptosis^7^. So, we assessed the impact of cisplatin on that pathway and suggested a role of iron chelator hindering neurotoxic pathway.

Our results showed that the given deferiprone dosage starting three days before and continuing four days after cisplatin dosage as if enveloping the cisplatin and hindering its neurotoxicity. In the same way, firstly, the nerve functions were evaluated and showed significant improvement in the physiological function as represented by decreased latency of withdrawal reflex back to normal in hot plate, cold allodynia, and tail flick tests. Also, the adhesive tape test contact and removal results have been improved. However, regarding adhesive tape contact not reaching the control group while the removal test reaching the control, this might have been attributed to the more damaging effect on sensory than motor nerve fibers. Also, deferiprone improved NCV in sciatic nerve significantly reaching the control group results.

Secondly, improved nerve tissue histopathological change in the form of apparently normal bundles of nerve fibers surrounded by perineurium and covered by epineurium containing arterioles and venules. Multiple nerve fibers, endoneurium, perineurium and venules were seen in addition to Multiple myelinated, multiple myelinating nerve fibers and multiple Schwann cells. The silver immune-staining indicated neurofibrils in multiple nerve fibers with multiple Schwann cells that was confirmed by PNCA immunostaining. Also, a significant increase was recorded in the mean count of myelinated fibers and the mean area% of neurofibrils versus the Cis group with a significant increase in mean area of Silver and PCNA stained Schwan cells and nuclei versus the other groups. This indicated improving activity of these cells highlighting the positive effect of Def dosage that started before and continued after Cis dosage. The deferiprone effect on peripheral nerve has been deficient in the literature. Most of the studies detected its protective effect on brain and retinal neurons^87–92^. Also, Rayatpour and his colleagues showed its myelinating effect in optic nerve^77^.

Thirdly, we referred this improvement to the hindering of ferritinophagy in nerve tissues, in which ferritinophagy inducers were down regulated while suppressors were upregulated. This was accompanied by reduced iron dependent lipid peroxidation (MDA) that stamps ferroptosis and improves the glutathione antioxidant system (GSH, GPX4 and SLC7A11), the main regulator counteracting ferroptosis^9,10,93^. Overexpression of GPX4 could inhibit ferroptosis induced by cisplatin^9,10,52,94,95^. Iron chelators improved glutathione antioxidant system^96,97^. It was detected that deferiprone could inhibit ferroptosis through the regulation of iron levels^7,22,65,98^. In our study deferiprone ameliorated ferritinophagy by down regulation of NCOA4 and IREB2 together with upregulation of FTH1^99^. Inhibiting NCOA4 or its down regulation hinders ferritinophagy and interferes with ferroptosis^100–104^. Inhibition of IREB2 could increase FTH1 expression accordingly inhibit ferroptosis^105^. Rayatpour group suggested an anti-ferroptotic effect of deferiprone in the optic nerve damage in vivo and in vitro through improving intracellular iron load and oxidative stress^77^.

## Conclusion

In conclusion, this study demonstrates that cisplatin dosage with deferiprone significantly ameliorates cisplatin-induced polyneuropathy in a rat model. Our findings reveal that this neuroprotective effect is mediated through the modulation of the ferritinophagy pathway. Deferiprone treatment effectively downregulated ferritinophagy inducers (NCOA4, IREB2) and upregulated inhibitors (FTH1, GPX4, SLC7A11), in addition resulting in reduced iron-dependent lipid peroxidation and improved glutathione antioxidant system function. The combined treatment led to significant improvements in nerve physiological functions, including better sensory and motor responses, enhanced nerve conduction velocity, and improved histological integrity of peripheral nerves. These improvements were associated with increased myelination, reduced Schwann cell apoptosis, and enhanced neurofilament preservation.

While these results are promising, it is crucial to note that further research is needed to optimize the dosing regimen and to investigate potential interactions between deferiprone and cisplatin’s anti-tumor efficacy. Future studies should focus on translating these findings to clinical settings, exploring the long-term effects of this combination therapy, and evaluating its efficacy in various cancer types and stages. This novel approach of using an iron chelator to mitigate chemotherapy-induced neurotoxicity opens up new avenues for improving the quality of life of cancer patients undergoing cisplatin treatment. It also underscores the importance of targeting iron homeostasis and ferritinophagy in neuroprotective strategies against chemotherapy-induced peripheral neuropathy.

## Limitations

In this study, only one dosage of the drugs (cisplatin and deferiprone) was used while trying further dosages and mechanisms of administration would be considered in next funded project for further investigations.

## Electronic supplementary material

Below is the link to the electronic supplementary material.

Supplementary Material 1

## Data Availability

All data generated or analyzed during this study are included in this published article [and its supplementary information files].

## Abbreviations

Cis: Cisplatin
Def: Deferiprone
GSH: Glutathione
IP: Intraperitoneal
IREB2: Iron responsive element binding protein 2
MDA: Malondialdehyde
NCV: nerve conduction velocity
NCOA4: Nuclear receptor coactivator 4
SCs: Shwan cells
SLC7A11: Solute carrier family 7 member 11 (system x^−^ _c_)
ROS: Reactive oxygen species
