# Supplementary material for: Deferiprone ameliorates cisplatin induced peripheral neurotoxicity via ferritinophagy adjustment
Source: Sci Rep. 2025 Feb 6;15:4485. doi: 10.1038/s41598-025-87628-x (PMC11802739; doi:10.1038/s41598-025-87628-x)
Supplement: Supplementary file 1 — Supplementary Material 1 [file 41598_2025_87628_MOESM1_ESM.pdf]

Supplementary data

Table 1: Results of Physiological function assessment tests Mean ± Standard Deviation ± Standard Error

Physiological function assessment (Mean ±SD±SE)	Control	Def	Cis	Cis-Def
1. Adhesive tape contact	7.400 ±5.413±2.421	8.500 ±3.416±1.708	162.7 ±30.02±17.33	40.00 ±7.000±4.041
2. Adhesive tape removal	48.25 ±16.09±8.045	40.25±4.992±2.496	177.0 ±5.196±3.000	39.25 ±5.909±2.955
3. Tail flick test	5.733 ±1.861±1.074	3.575 ±1.261±0.6303	18.00 ±6.595±2.950	3.833 ±2.229±0.9098
4. Hot plate test	5.267 ±1.877±1.084	8.667 ±2.082±1.202	23.00 ±6.928±4.000	9.750 ±3.775±1.887
5. Cold allodynia test	8.500 ±4.435±2.217	13.75 ±4.425±2.213	33.75 ±8.057±4.029	7.200 ±2.588±1.158
6. Rota road test	4.190 ±0.8307±0.4796	3.030 ±0.9554±0.5516	2.480 ±0.6350±0.3666	4.000 ±1.732±1.000
7. Sciatic nerve (Motor)	69.74 ±2.242±0.9154	70.54 ±2.956±1.207	47.37 ±7.062±2.883	65.75 ±6.232±2.544
8. Sciatic nerve (Sensory)	71.44 ±0.9692±0.3957	71.19 ±2.631±1.074	58.09 ±5.742±2.344	65.69 ±3.622±1.479

Table 2: Results of Biochemical markers assessment tests using ELISA or quantitative RT-PCR Mean ± Standard Deviation ± Standard Error

Biomarker assessment using PCR and ELISA	Control	Def	Cis	Cis+Def
1. MDA	0.2854 ±0.0408±0.02041	0.3175 ±0.02541±0.01270	1.655 ±0.06357±0.03179	0.6778 ±0.2105±0.1052
2. GSH	1.673 ±0.1069±0.05344	1.468 ±0.1424±0.07122	0.4470 ±0.08963±0.04481	1.578 ±0.06185±0.03092
3. GPX4	1.067 ±0.1034±0.05168	1.010 ±0.2529±0.1265	0.1698 ±0.05138±0.02569	0.9629 ±0.1807±0.09033
4. SLC7A11	1.111 ±0.1017±0.05085	1.017 ±0.1681±0.08405	0.2535 ±0.09390±0.04695	0.8629 ±0.1691±0.08454
5. NCOA4	1.045 ±0.09709±0.04855	1.075 ±0.1215±0.06073	3.976 ±0.5613±0.2806	1.778 ±0.1287±0.06435
6. IREB2	0.9890 ±0.1511±0.07554	1.124 ±0.1617±0.08086	4.353 ±0.8156±0.4078	1.666 ±0.1761±0.1017
7. FTH1	1.040 ±0.1867±0.09333	1.116 ±0.1262±0.06308	0.1720 ±0.07873±0.03937	0.8918 ±0.1349±0.06747
